# Deep learning in macroscopic diffuse optical imaging

**DOI:** 10.1117/1.JBO.27.2.020901

**Published:** 2022-02-25

**Authors:** Jason T. Smith, Marien Ochoa, Denzel Faulkner, Grant Haskins, Xavier Intes

**Affiliations:** a Rensselaer Polytechnic Institute, Department of Biomedical Engineering, Troy, New York, United States; b Rensselaer Polytechnic Institute, Center for Modeling, Simulation and Imaging for Medicine, Troy, New York, United States

**Keywords:** macroscopic diffuse optics, deep learning, diffuse optical tomography, review, diffuse optics, fluorescence molecular tomography, lifetime imaging, tissue hemodynamics

## Abstract

**Significance:**

Biomedical optics system design, image formation, and image analysis have primarily been guided by classical physical modeling and signal processing methodologies. Recently, however, deep learning (DL) has become a major paradigm in computational modeling and has demonstrated utility in numerous scientific domains and various forms of data analysis.

**Aim:**

We aim to comprehensively review the use of DL applied to macroscopic diffuse optical imaging (DOI).

**Approach:**

First, we provide a layman introduction to DL. Then, the review summarizes current DL work in some of the most active areas of this field, including optical properties retrieval, fluorescence lifetime imaging, and diffuse optical tomography.

**Results:**

The advantages of using DL for DOI versus conventional inverse solvers cited in the literature reviewed herein are numerous. These include, among others, a decrease in analysis time (often by many orders of magnitude), increased quantitative reconstruction quality, robustness to noise, and the unique capability to learn complex end-to-end relationships.

**Conclusions:**

The heavily validated capability of DL’s use across a wide range of complex inverse solving methodologies has enormous potential to bring novel DOI modalities, otherwise deemed impractical for clinical translation, to the patient’s bedside.

## 1 Introduction

The scientific value of monitoring biological tissues with light was recognized many centuries ago, as reported in published works dating as early as the late 1800s for monitoring brain hemorrhage, ^1^ as well as the early 1900s for imaging breast cancer^2^^,^^3^ and performing tissue oximetry.^4^ Since then, optical imaging techniques have greatly benefited numerous biomedical fields. Particularly, a wide range of optical techniques provide unique means to probe the functional, physiological, metabolic, and molecular states of deep tissue noninvasively with high sensitivity. As scattering is the predominant phenomenon ruling light propagation in intact biological tissues, the photons harnessed to probe the tissue have typically experienced multiple scattering events (or diffusion); therefore, this field can be broadly classified as diffuse optical imaging (DOI). Applications of DOI range from macroscopic extraction of optical properties (OPs), such as absorption and scattering, for further tissue classification and 2D representations,^5^^,^^6^ to 3D tomographic renderings of the functional chromophores or fluorophore within deep tissues.^7^^–^^10^ Despite the numerous benefits of DOI, its diverse implementations can still be challenging due to the necessity of computational methods that model light propagation and/or the unique contrast mechanism leveraged to be quantitative. Hence, numerous implementations in DOI require a certain level of expertise while also being dependent on the optimization of intrinsic parameters of these computational models—limiting their potential for dissemination and, hence, translational impact.

Meanwhile, over the last decade, the implementation of data processing methodologies, namely deep learning (DL), promises the development of dedicated data-driven, model-free techniques with robust performances and user-friendly employability. DL methods are increasingly utilized across the biomedical imaging field, including biomedical optics.^11^ For instance, molecular optical imaging applications from resolution enhancement in histopathology,^12^ super-resolution microscopy,^13^ fluorescence signal prediction from label-free images,^14^ single-molecule localization,^15^ fluorescence microscopy image restoration,^16^ and hyperspectral single-pixel lifetime imaging^17^ have been enhanced by recent developments in DL. Following this trend, DL methodologies have also been recently used for DOI applications. In this review, we provide a summary of these current efforts. First, we introduce the basic technical concepts of DL methods to be addressed and the commonly employed frameworks. The subsequent sections will describe the architectures developed or adapted for different macroscopic DOI applications, including 2D retrieval of OPs, macroscopic fluorescence lifetime imaging (MFLI), single-pixel imaging, diffuse optical tomography (DOT), fluorescence, and bioluminescence molecular tomography.

## 2 General Overview of Deep Learning Frameworks

This section briefly overviews technical DL concepts that are addressed throughout the article. For those new to DL, the authors suggest prior reading for maximized accessibility of the topics discussed herein.^18^^,^^19^ Also for readers more interested in the mathematical links between classical optical computational image formation and DL methods, we refer them to Ref. 20.

DL is a special class of machine learning (ML) algorithms that incorporate multiple “hidden layers” (i.e., layers other than input and output) aimed at extracting latent information (commonly referred to as “features”) of higher and higher levels of abstraction/nonlinearity (interactive visual supplement available elsewhere^21^). Such an approach was proposed as early as 1943 by Pitts and McCulloch,^22^ who developed a computer model inspired from the human brain neural network. This development was followed by the implementation of models with polynomial activation functions, aimed at inducing nonlinear relationships between output and input/set of inputs and carrying forward of the best statically features to the next layers, by Ivakhnenko.^23^ Then Fukushima and Miyake^24^ reported on the first “convolutional neural network” (CNN), neocognitron, that was based on a hierarchical, multilayer architecture. These concepts were improved upon by the incorporation of backpropagation methodologies during model training. LeCun^25^ combined DL with backpropagation to enable the recognition of handwritten digits. Meanwhile, computational power was steadily increasing with the critical development of GPUs (graphics processing units). The adoption of GPUs enabled the development of fast DL models that were computationally competitive with other ML techniques such as support vector machines (SVM)^26^ and linear/logistic regression. Since then, DL has known continued growth with the notable development of ImageNet,^27^ which heralded the pairing of DL and big data. With the increased computing speed, it became clear that DL had significant advantages in terms of efficiency and speed. In particular, the computer vision community has embraced the use of CNNs after the breakthrough results of AlexNet^28^ in the large-scale visual recognition challenge (ILSVRC) of 2012. Since then, a large variety of models exhibiting state-of-the-art performance have been developed and increasingly improved upon for countless applications in computer vision—including classification, object detection, and segmentation. Today, when designing a DL-based solution to a given problem, consideration should be given to the type of network that is chosen, that network’s architecture, and the way in which the network is trained. The remainder of this section provides a layman introduction for these three key elements.

### 2.1 Neural Network Types

The simplest form of artificial neural networks (ANN) still commonly employed is multilayer perceptron (MLP). The network architecture of an MLP is composed of at least three perceptron layers: an input layer, a hidden layer, and an output layer. After the input layer, each node of the MLP is passed through a nonlinear activation function selected *a priori*. The combination of multiple layers and nonlinear activations enables MLPs to compute nontrivial problems using only a small number of nodes.^29^ In MLPs, all neurons in a layer are connected to all activations in the previous layer, each of which is referred to as a fully connected layer (FC layer). This FC nature can become a disadvantage as the total number of parameters can grow extremely large with increased model depth (number of hidden layers) and/or width (number of neurons at each layer). For instance, an MLP designed for a modestly sized 2D image input will possess many parameters, which is problematic for both increased overfitting potential and memory limitations.^30^ Moreover, many applications of interest in biomedical imaging have two or more dimensions. The need to flatten these images into 1D input for MLPs often leaves achieving even modest levels of spatial equivariance computationally problematic. Indeed, MLPs inherently lack the capability to model even the most simplistic of translational invariance without many hidden layers. Hence, to use MLPs for these applications, DL practitioners would need to walk a fine line between a model with too little parameters (spatial invariance) and a model with too many parameters (prone to overfitting, computational inefficiency, etc.).^31^ In contrast, CNNs provide a much higher degree of translational invariance and are capable of highly sensitive, localized, and computationally efficient feature extraction by way of their very design. Hence, the use of MLPs for image formation has been largely succeeded by CNNs in most applications. CNNs are neural networks that use convolution in place of general matrix multiplication in at least one of their layers. Similar to MLPs, CNNs are comprised on an input layer, hidden layers, and an output layer. These hidden layers typically consist of convolutional layers that pass sequentially the convolution of their input to the next layer (with other type of layers such as pooling layers, FC layers, and normalization layers). The nature of the convolution operation allows for reducing the number of learnable parameters necessary for image-based feature extraction and, hence, increasing the depth of the network architecture.

The size of the set of output feature maps following each convolutional layer depends upon the number of kernels used, the size of the kernel used, and the stride associated with the sliding convolution. Along with providing the network with translation equivariance, zero padding can be used to provide further control over the dimensionality of output feature maps and allows for the size of the feature maps to be preserved after the convolutional operation. This is useful for element-wise combinations of feature maps in which the sizes of the sets of feature maps must be identical.

Although downsampling can be performed using convolutions without zero padding, this may not be ideal for some applications. A common strategy for reducing the size of a set of feature maps is pooling. In particular, max pooling is the most popular pooling strategy because it is both computationally inexpensive and mostly translationally invariant. Additional pooling strategy alternatives exist, such as global average pooling, which has demonstrated increased performance in applications of implicit object localization.^32^

Moreover, the previously discussed convolutional and FC layers perform linear operations. Therefore, because a composite function of linear functions is still a linear function, neural networks that are solely composed of these layers would be unable to approximate a nonlinear function. Thus nonlinear activation functions are used to insert nonlinearity into the convolutional and FC layers such as ReLU, Leakly ReLU, ELU, PreLU, Tanh, Softmax, and Sigmoid functions. In addition, recent work has demonstrated promising results using more generalized and intuitive activation functions, such as GenLU.^33^

One limitation of traditional CNNs is their use of FC-layers in their architecture, making them not well suited when processing high-resolution images, for instance. Conversely, fully convolutional networks (FCNs)^34^ used for image formation, such as convolutional autoencoders, do not contain “dense” layers (i.e., FC-layers). Instead, an FCN utilizes 2D convolutions that perform the feature extraction and mapping task of FC-layers in conventional CNNs. Hence, FCNs can make inferences in high-dimensional spaces but are also uniquely amenable to input of variable sizes. FCNs have exhibited state-of-the-art performance for many computer-vision tasks, especially when dense labeling is required.^34^^–^^36^ FCNs also have the advantage of providing end-to-end solutions (execute a series of tasks as a whole). Specifically, autoencoder structures that are mentioned in this text are FCNs that have an output layer of equal size to the input layer and consist of an encoder and a decoder section. The encoder transforms the input into a specific set of features, and these features can then be interpreted by the decoder section to recover the original data.^37^

However, in CNNs, the output is produced with the underlying assumption that two successive data inputs are independent of each other. In other words, they do not have “memory,” and their output is independent of previous element in a sequence. Recurrent neural networks (RNNs) have been specifically developed to model/process time series data (e.g., video sequences). Each element (image) in the time series data is mapped to a feature representation, and the “current” representation is determined by a combination of the previous representations and the “current” input datum. In other words, RNNs have loops between layers that allow information to persist. One issue often encountered when using RNN is the vanishing/explosion gradient problems (difficulty in training network). Long short-term memory networks have been designed to overcome this issue and are widely used in classification and forecasting based on time series input across many applications.^38^

### 2.2 Considerations in Network Architecture

Deep neural networks (DNNs) are now consistently producing the state-of-the-art results in countless applications across fields. Beyond the refinements in network architecture and training methodologies (see the next section), it is unquestionable that the computational prowess of current GPU units in conjunction with the availability of large datasets are central to these successes. Current DL implementations have been characterized by an increase in depth and computational complexity. For instance, the visual geometry group demonstrated that the depth of a network was a critical component to achieving better classification accuracy in CNNs.^39^ However, a phenomenon known as “degradation” was observed when network depth was increased. Degradation refers to the sudden rapid deterioration of network performance during training. One of the issues associated with increasing network depth is the explosion or vanishing of gradients during backpropagation. To address this challenge, Ioffe and Szegedy^40^ introduced “batch normalization.” Batch normalization layers are used to fix the mean and variance of layer output during forward passing, squashing any large activations and increasing network stability. The mechanism of how batch normalization works has been largely accepted as being due to the reduction of internal covariate shift or abrupt changes in the distribution of the layer input. Santurkar et al.^41^ recently illustrated that this was not so and that more exploration is needed for a definitive answer. Because the effect of batch normalization is deemed dependent on the batch size and sometimes is misleading to use it for recurrent networks layer normalization has been proposed for use instead.^42^ In this case, the normalizing mean and variance are calculated from all inputs to neurons in a layer per each sample. It has been shown to be easier to implement in recurrent networks and to further reduce training time. In this regard, weight normalization^43^ can also be applied successfully to recurrent models and has shown improved speed compared with batch normalization. The use of normalization techniques coupled with a good weight initialization strategy^44^ can be a key to avoiding degradation and accomplishing network convergence.

**Fig. 1:**
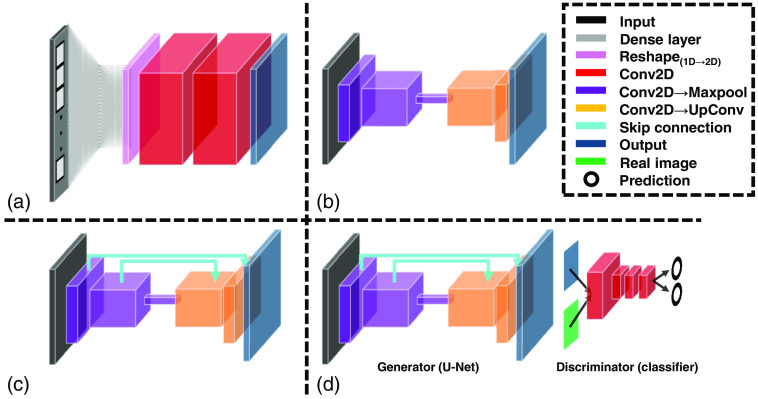
(a) AUTOMAP, an example network architecture capable of mapping 1D sensor-domain input to 2D image space through a combined use of FC and convolutional layers. (b) Classical encoder–decoder convolutional network architecture. (c) U-Net: variation of the encoder–decoder architecture by adding long skip connections to help deeper networks avoid suboptimal convergence (e.g., vanishing gradient) and to recover information lost during encoder downsampling. (d) GAN framework with U-Net as generator. The “discriminator” is trained to discriminate between “real” and “fake” (i.e., ground truth and GAN-generated, respectively) image data.

As an example to address the degradation effect, a DNN framework deemed “inception” developed by Szegedy et al.^45^ utilized feature concatenation of activation layers to develop larger networks than had been viable without performance degradation. Conceptually, the group’s work stemmed from the idea that visual information should be processed at different scales and aggregated to enable subsequent layers to utilize information from several scales concurrently. The group’s model architecture (deemed “GoogleLeNet”) was the first of its kind to increase network depth and width without increasing computational burden, and it allowed the group to achieve first place in the ILSVRC 2014 classification challenge by a significant margin. In addition, DNN “ResNet,” along with the concept of a “residual block,” was proposed by He et al.^46^ The principal contribution of this work was demonstrating that residual connections (element-wise sums of a set of feature maps) can be used to mitigate the effect of vanishing gradients and improve training stability—even in CNNs of a previously inconceivable number of hidden layers. The idea is that, if added model depth could compromise performance, residual blocks will converge to identity mapping of the earlier layer’s output. Ronnenberg et al.^47^ proposed U-Net, which used ideas from inception and employed concatenations instead of residual connections to combine features learned from early layers with more abstract features extracted at deeper layers for semantic segmentation. Given both its demonstrable performance and adaptability, this architecture has been widely adopted, and several of its extensions have exhibited state-of-the-art performance across a great number of applications. Another important consideration when designing networks is to ensure that the proposed architecture is secure and not tricked by adversarial attacks that might want to disrupt the network’s estimations. Generative adversarial networks (GANs) are a unique class of CNN capable of being used in supervised, unsupervised, or reinforcement learning (RL)-based applications. The defining feature of a GAN is its “discriminator”—an extension of the traditional CNN [deemed the GAN’s “generator,” Fig. 1(d)], which conventionally acts as a classifier.^48^ Indeed, the role of a GAN’s discriminator is to discern “real” data (i.e., ground truth) from “fake” data (i.e., the generator CNN’s output). In practice, this is actualized through the incorporation of an additional loss term associated with the discriminator that aims to update the discriminator’s weights in such a way that the discriminator becomes progressively more proficient at telling the difference between the CNN’s output and that used for ground truth. Hence, conventional loss metrics (e.g., MSE, MAE, and SSIM) are augmented by an additional discriminator loss that guides the model further toward generating data with statistics equivalent to that of the target output.^49^ This loss often takes the form of binary cross entropy—a loss used to map each reconstruction to two values between one and zero (i.e., one-hot encoding). These values act as the model’s predicted likelihood of each reconstruction being real or produced by the model. Hence, the aim is to gradually “fool” the discriminator, where it would eventually be unable to discriminate between the model’s output and ground-truth data.

Overall, the goal of designing a neural network is to maximize performance while minimizing the resources needed to train this network. Indeed, there is a current recognition that many of these DL systems train models that are richer than needed and use elaborate regularization techniques to keep the neural network from overfitting on the training data. This comes at a high expense in the form of computing power, time, and the lack of global accessibility. This is where the concept of network interpretability^50^ becomes so important. Interpretability refers to how easy it is for a human to understand why the output and decisions made by an ML model are the way they are. The higher the interpretability of the model is, the easier it might be for a human to understand its design and improve on it.

### 2.3 Training Methodologies

DL methodologies are often classified based on the type of data and associated training approaches. These include supervised, semisupervised, unsupervised, reinforcement, and adversarial learning. We provide below a brief summary of these different learning approaches. Supervised learning is the most utilized learning strategy in ML. It relies on having both training and validation datasets along with their “ground-truth” complement. The ground truth is unique to each application (e.g., one-hot encoded vectors for image classification) and implies that the relationship between input and output for is known explicitly before training. Model training (i.e., gradual updates to a network’s weights) is performed through minimization of a cost function (often referred to as “loss”) via the process of backpropagation. Backpropagation, the fundamental method with which neural networks use to “learn,” makes use of the chain rule to propagate updates throughout the network topology in a way that further minimizes the chosen cost function. For instance, in the case of supervised learning, the cost function inversely represents the accuracy between the model prediction and known ground truth for all training inputs. For example, under a supervised framework, a network that classifies images is trained using classifications that are made by practitioners. In this application, humans are perfectly capable of generating trustworthy labels. For example, practitioners can of course be trusted to correctly classify a picture of a dog as “dog.” However, supervised learning has its limitations in applications that are more difficult because human labels are often costly with respect to both time and resources. Further, depending on the target application, given labels may not be trustworthy or cannot be generated. In this case, one can consider unsupervised learning.

Unsupervised learning aims to discover unknown relationships or structure in the input data without human supervision (or minimal). The appeal of unsupervised learning is that many applications do not yet benefit from large datasets that have been exhaustively labeled. Unsupervised DL methods include clustering, sample specificity analysis, or generative modeling. Semisupervised learning and weakly supervised learning, which will be described when discussing the specific works that use them, are considered hybridizations of supervised and unsupervised learning strategies. A particularly unique unsupervised learning strategy that has been employed in applications ranging from unmanned robotic navigation to defeating chess grandmasters is RL.^51^ Problems that use RL can essentially be cast as Markov decision processes associated with a combination of state, action, transition probability, reward, and discount factor. When an agent (i.e., the decision maker) is in a particular state (e.g., location in a maze), it uses a policy to determine which action to take among a set of possible actions. The agent then undergoes the policy-informed action and transitions into the next state, where a reward value corresponding to desirability of the new state is obtained. This process repeats until an end point is reached (e.g., reaching the exit of a maze). Afterward, a total cumulative reward value is calculated and used as feedback for parametric adjustments and subsequent iterations, gradually guiding the agent to “learn” which actions are “good” and “bad” at each state. In this way, the agent is made to maximize the total reward that it receives while performing a given task. The primary objective is to learn the optimal policy with respect to the expected future rewards. Instead of doing this directly, most RL paradigms learn the action-value function using the Bellman equation. The process through which action-value functions are approximated is referred to as “Q-learning.”^52^ These approaches utilize action-value functions that determine the advantageous nature of a given state and state-action pair, respectively. Further, an advantage function determines the advantageous nature of a given state-action pair relative to the other pairs. In recent years, these approaches have demonstrated remarkable feats, famously achieving superhuman performance when applied to various board/video games such as Go^53^ and StarCraft.^54^ At present, the applications of RL, though undeniably laudable and continuously expansive, have been somewhat limited in scope given the narrow subset of pressing problems for which the use of RL would be uniquely advantageous relative to the approaches previously discussed.^55^ Another important methodology is adversarial learning,^56^ which is an ML method that involves the use of adversarial samples that closely mimic “correct” inputs with a main purpose of tricking the model and yielding incorrect predictions. Understanding adversarial attacks is highly important for the design of secure ML models.

## 3 Deep Learning for Estimating Optical Properties

Investigating not only tissue structure but also its functional status through light–matter interactions has been the focus of multiple biomedical optics applications over the last few decades. Of importance to the field, most tissue types and/or diseases to be monitored are relatively deep seated *in vivo*, i.e., below the epithelial layers. For such tissues, with depth ranging from a few hundred microns to a few centimeters deep, DOI techniques are required as the collected photons have experienced multiple scattering events even at the typically longer wavelength employed.^57^^–^^59^ DOI encompasses various techniques that aim to quantify the tissue OPs that govern light propagation at this spatial scale,^58^^,^^60^ namely the absorption coefficient $\mu_{a}$, or its related chromophores when spectral information is available (including oxy-, deoxy-hemoglobin, water, and lipids^59^), and the scattering coefficient $\mu_{s}$ (or typically its isotopic equivalent, the reduced scattering coefficient $\mu_{s}^{\prime }$). Such an estimation task is typically performed by fitting the experimental data to a dedicated mathematical model. Hence, it was recognized early on that neural network models could perform such tasks. The first use of neural networks to infer OPs was demonstrated by Farrell et al.,^61^ who constructed an ANN that was able to retrieve OPs from spatially resolved reflectance data. This ANN was designed to output the reduced scattering coefficient $\mu_{s}^{\prime }$ and the absorption coefficient $\mu_{a}$, while being inputted with a transform of the diffuse reflectance profile R based on radial distance ρ to emphasize its relationship with the total optical transport coefficient $\mu_{t}^{\prime }$. The ANN was composed of eight input nodes (eight radial separations in the reflectance data), a hidden layer of eight nodes, and an output layer with two nodes—one each for the effective and the total transport coefficients. To train the network, reflectance datasets were acquired from various well-characterized materials. Overall, the authors reported that their network was able to output results within <7% root-mean-square error while using an unlabeled test set. Additionally, this ANN proved 400 times faster than the conventional gradient search algorithm by Bevington in their time.^61^ The high accuracy and fast computational speed of this early work highlight the potential of neural network for diffuse optical spectroscopy. This seminal work has recently been followed by more contemporary implementations that benefit from the exponential computational power increase achieved since. Gökkan and Engin^62^ developed an ANN that, similar to Farrell et al., was designed to estimate the absorption and scattering coefficients directly but using 17 spatially resolved data extracted from a dense reflectance measurement acquired via a CMOS camera. The ANN was trained using the Monte Carlo dataset and tested on liquid phantoms with properties ranging from $\mu_{a}\in [0.01\text{ to }12]\;{\mathrm{cm}}^{-1}$ and $\mu_{s}^{\prime }\in [5\text{ to }35]\;{\mathrm{cm}}^{-1}$. These OPs cover the wide range of *in vivo* conditions. The author reported a good agreement between the liquid OP’s values and the ANN estimation, though they did not provide quantitative accuracy values. Ivančič et al.^63^ developed another ANN to estimate tissue OPs, but for a more challenging cases of 4 parameters: $\mu_{a}$, $\mu_{s}^{\prime }$, subdiffusive reflectance first similarity parameter (γ), and next similarity parameter (δ). These additional parameters significantly affect the reflectance profile when operating in a small spatial range in which the photon collected can be minimally scattered, and hence, the reflectance patterns still greatly depend on the anisotropic characteristics of the scattering interactions. This is the first neural network implementation reported to estimate OPs beyond $\mu_{a}$ and $\mu_{s}^{\prime }$. Of note, a separate ANN was used to estimate each optical parameter individually. Each ANN was comprised of an input layer, two hidden layers, and an output layer with a variable number of hidden nodes. The input data consisted of five reflectance source–detector separations (220, 440, 660, 880, and 1200  μm), and the light propagation was validated with a Monte Carlo simulation. The ranges of OPs considered were $\mu_{a}\in [0.0005\text{ to }0.25]\;{\mathrm{mm}}^{-1}$ and $\mu_{s}^{\prime }\in [0.5\text{ to }2.0]\;{\mathrm{mm}}^{-1}$. The authors compared their results with spatially resolved reflectance data from a hyperspectral source and an optical fiber probe with the best results achieved when using the hyperspectral source. They observed a root mean squared error with 1.0%, 1.3%, 1.1%, and 4% for $\mu_{a}$, $\mu_{s}^{\prime }$, γ, and δ, respectively. Moreover, their ANN approach was four orders of magnitude faster than the lookup table (LUT) method that they used as a benchmark.

These works highlight the potential of neural networks for OPs estimation, especially for fast inference. In all cases, reflectance geometry was used as it is the most useful sensing method in clinical scenarios. Still, recent progress in structured light imaging has led to a popular new technique to estimate the OPs of tissue over large field of view and in real time, namely, spatial frequency domain imaging (SFDI).^64^

### 3.1 SFDI-Based Optical Properties Classification with Deep Learning

OPs retrieval on a large field of view is mainly motivated by identifying pathologic tissue areas (burn degree assessment, malignant tumor versus benign, etc.). Hence, the OPs are used for the classification task. This can be performed using ML methods that have been developed over the last three decades, including SVM^26^^,^^65^ or random forest (RF),^66^ which are referred to as shallow learning techniques. For instance, Laughney et al.^67^ utilized intraoperative SFDI paired with a k-nearest neighbor (KNN) algorithm analyses for classifying tissue as benign or malignant before conventional tissue resection in human patients undergoing lumpectomy. More recently, Rowland et al.^68^ performed multifrequency, multiwavelength SFDI along with SVM classification to discriminate between types of controlled burns *in vivo* (porcine subjects). Altogether, the use of shallow learning models for these example cases illustrated good classification performances. However, though shallow supervised learning classification techniques can provide a higher degree of interpretability than DL, they are not optimal when the data are high dimensional in nature (such as an image). In such cases, DL models have been reported to consistently outperform these shallow learning methods. Hence, the current thrust in the field is to craft dedicated DL models and, when possible, benchmark them against well-established ML methods.

For instance, Sun et al.^69^ employed SFDI for detection of early fungal infection in peaches while performing the classification task using partial least-squares discriminative analysis (PLSDA) and a CNN-based workflow. Maximum detection accuracies using PLSDA analyses reached 84.3% compared with a CNN, trained with a small fraction of the total data collected, which reached 98.6% and 97.6% detection accuracy for the two most challenging cases. However, as Li et al.^70^ recently demonstrated, utilizing a boosted ensemble of shallow classifiers can also provide high-level discriminative performance as well as enhanced robustness. Embracing this approach, Pardo et al.^71^ recently presented a DL routine that utilizes an ensemble of DNNs, each of which is crafted to take a different sized image patch as input, as an approach to perform patch-wise tissue classification for lumpectomies via SFDI.^72^ Therein, the authors self-developed method of “self-introspective learning,”^73^ which was an intrinsic measure of the trained model’s familiarity with the input upon inference, was incorporated. Pardo et al.^74^ extended this work via the use of adversarial learning. With the incorporation of an autoencoder for data dimensionality reduction, the group developed an unsupervised method for real-time margin assessment of resected samples imaged with SFDI. The authors designed a “four-domain” approach, allowing for a large degree of network interpretability. Indeed, for successful clinical translation of classification algorithms, both optimal model performance as well as capability to provide insight into the models’ decision-making process to the end user will be necessary. Still, the models described above are dedicated to a specific classification task that can be application specific but also typically requiring preprocessing of the experimental datasets to provide the required inputs. Nevertheless, DL methods are also well-suited for tackling the SFDI inverse problem that aims at retrieving the wide-field OPs from experimental spatial modulation transfer functions.

### 3.2 SFDI-Based Optical Properties Reconstruction with Deep Learning

**Fig. 2:**
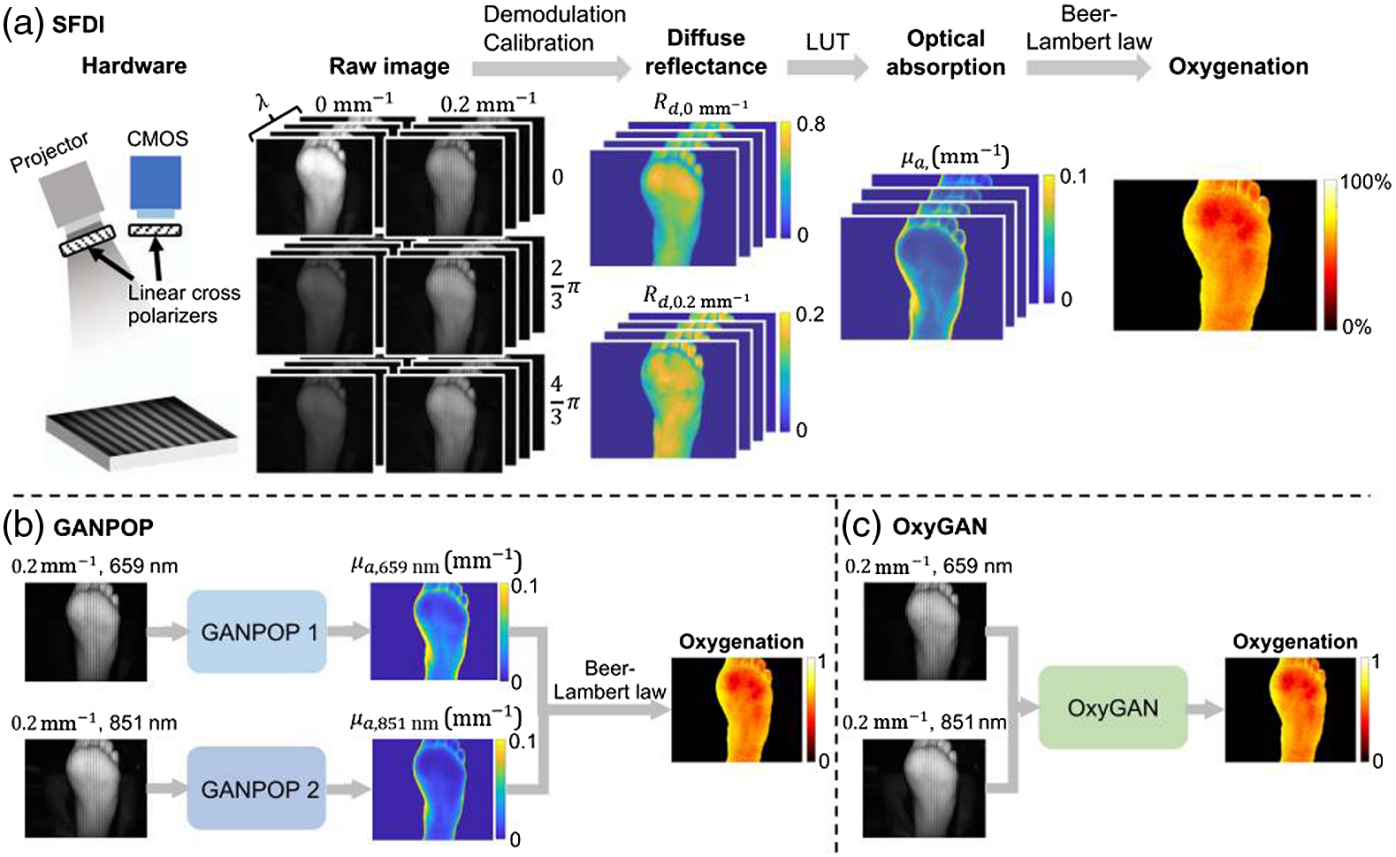
(a) Traditional method for retrieving oxygenation values via SFDI. (b) GANPOP and (c) OxyGAN (reproduced with permission from Ref. 78).

Estimating the OPs in SFDI typically involves an inverse problem that includes an optical forward model.^64^ This can be performed via iterative fitting using analytical or stochastic approaches (Monte Carlo) or LUTs. Even if effective, these approaches can require some level of expertise and can be computationally burdensome such that they do not lend themselves to real-time applications, a main feature of SFDI appeal. AI-based models are expected to significantly speed up the OPs estimation speed of the SFDI while potentially reaping benefits such as robustness to noise. In this regard, Panigrahi and Gioux^75^ attempted to tackle this bottleneck via an RF approach. Though the group reported reduced accuracy using RF compared with even the low-density LUT, the authors noted that the investigation was limited to the use of just two spatial frequencies. Thus the authors concluded that other ML techniques, especially DL, may be better suited for time-sensitive analyses of data containing multiple spatial frequencies. Zhao et al.^76^ published the first work applying DL to SFDI for OPs retrieval via deep MLPs. Notably, the group trained their model using sparsely sampled, MC-simulated data and validated their approach on human cuff occlusion data *in vivo*—exhibiting significant increases in computational speed as well as comparable accuracies with state-of-the-art iterative solvers. Building on this work, Zhao et al.^77^ employed a deep residual network (DRN) to go a step further and map SFDI-retrieved diffuse reflectance input to chromophore concentrations (${\mathrm{HbO}}_{2}$ and HHb) directly rather than to OPs. For this, the authors developed a simulation data routine for model training via pairing MC and Beer’s Law. Notably, upon *in vivo* validation, the authors’ DRN approach exhibited an order of magnitude speed boost versus the groups’ prior MLP. An alternative method aimed at decreasing the SFDI speed bottleneck in terms of data acquisition, single snapshot of optical properties (SSOP), attempts to decrease the number of acquisitions necessary to perform conventional demodulation to that of a single AC image [Fig. 2(a)].^79^^,^^80^ However, the method suffers from negative image artifacts that are intrinsic to the technique. These include blurred edges, frequency-dependent stripes, and decreased resolution, among others. Chen et al.^81^ reported the first DL technique aimed at performing SSOP reconstruction that demonstrated improved image quality. For this, the authors employed an end-to-end learning approach via a conditional GAN. The groups’ technique mapped multichannel input, comprised of target sample and calibration phantom images, directly to profilometry corrected OPs via a residual U-Net generator [Fig. 2(b)]. Alternatively, Aguénounon et al.^82^ recently developed an approach that instead used DL-based demodulation paired with GPU-accelerated computing for OP retrieval. Of significance, the authors focused on practical dissemination of the DL routine; because it does not require specific calibration for model output, the model complexity was made friendly to those without high-end GPUs, and profilometry was made to be an output of the model to provide greater insight into the model prediction. The group reported high-quality, profile-corrected OP retrieval across a 1024×1024 input in just 18.1 ms using a single NVIDIA GTX 1080Ti.

If retrieval of OPs across a wide FOV in real time is valuable, biological interpretation would still be greatly enhanced using multiple wavelengths for subsequent retrieval of chromophore concentrations directly. In this regard, real-time (1.6  ms/1024×1024 image) retrieval of both OPs and oxygenation maps has recently been accomplished by Aguénounon et al.^83^ through spatiotemporal modulation of two wavelengths coupled with a highly optimized computational framework—including LUTs employed via compute unified device architecture GPU processing. However, the authors’ optimized 2D-filtering technique introduced numerous artifacts to the retrieved image. Aiming to address this, Chen and Durr^78^ recently built upon their prior cGAN framework to map reference-calibrated, two-wavelength SSOP input directly to profile-corrected ${\mathrm{StO}}_{2}$ [Fig. 2(c)]. The authors, benchmarking against four-wavelength SFDI ground truth, reported markedly increased reconstruction accuracies compared with conventional SSOP solvers as well as their prior architecture paired with pixel-wise fitting. Notably, the trained generator was capable of 25 Hz ${\mathrm{StO}}_{2}$ retrieval across 1024×1024 FOVs using a quad-GPU workstation. Together, the authors’ results support the use of end-to-end DL solvers versus partial DL incorporation.

Given the above, successful adaptation of a DL-based workflow for real-time SFDI heralds its implementation in challenging clinical scenarios such as deployment for image guided surgery. Still, it is expected that further developments in DL models and training/validation strategies will continue to propel SFDI toward the patient bed side. Additionally, coupled with ever more sophisticated optical instruments, future work will likely focus on including the quantification of an increased number of relevant biological chromophores for improved specificity (oxy-hemoglobin, deoxy-hemoglobin, water, lipids, melanin, and yellow pigments). Beyond providing fast and robust image formation and classification tools, DL models are also expected to impact the next generation of SFDI instruments. Indeed, the successful implementations of relatively small DL models such as in prior work,^82^ enable their implementations on the back end of the instrumentation for clinically friendly form factors. Still, as in the field of DL for medical diagnosis at large, the black-box nature of current implementations limits their wide clinical dissemination. For clinical acceptance, explainable AI (xAI) methods that provide insight into the model’s decision-making will be critical for instilling confidence and widespread acceptance.^84^

## 4 Deep Learning for Macroscopic Fluorescence Lifetime Imaging

Fluorescence molecular imaging has been central to numerous discoveries and advances in molecular and cell biology. If fluorescence molecular imaging is still mainly performed using microscopy imaging techniques, mesoscopic and macroscopic fluorescence imaging have found great utility in imaging tissue at the organ and whole-body scales.^85^ Similar to nuclear imaging, fluorescence molecular imaging enables the probing of tissues beyond microscopy depth limitations with high sensitivity and specificity, with the advantage of a wide range of fluorescence probes, including fluorescent proteins, being commercially available, and with potential for efficient multiplexing (imaging multiple biomarkers simultaneously). Moreover, fluorescence imaging provides the opportunity to sense and quantify a unique contrast function: the fluorophore lifetime. Due to its high specificity and ability to monitor the molecular microenvironment and changes in molecular conformation as well as increasing commercial offering in turn-key imaging system, fluorescence lifetime imaging (FLI) has (re)gained popularity in the last decade. Still, FLI necessitates computationally expensive inverse solvers to obtain parameters of interest, which has limited its broad dissemination, especially in clinical settings. Hence, great interest in the last few years has been put into leveraging ML and DL models to facilitate image formation or classification tasks using this unique contrast mechanism. However, almost all of these works have been focused on applications in microscopy or raster-scanning based on time-resolved spectroscopy. Given that these works still provide relevant innovation and potential utility in future macroscopic FLI experiments, we include them in our summary below along with the existing work in ML applied to MFLI.

### 4.1 Deep Learning for Fluorescence Lifetime Image Classification

**Fig. 3:**
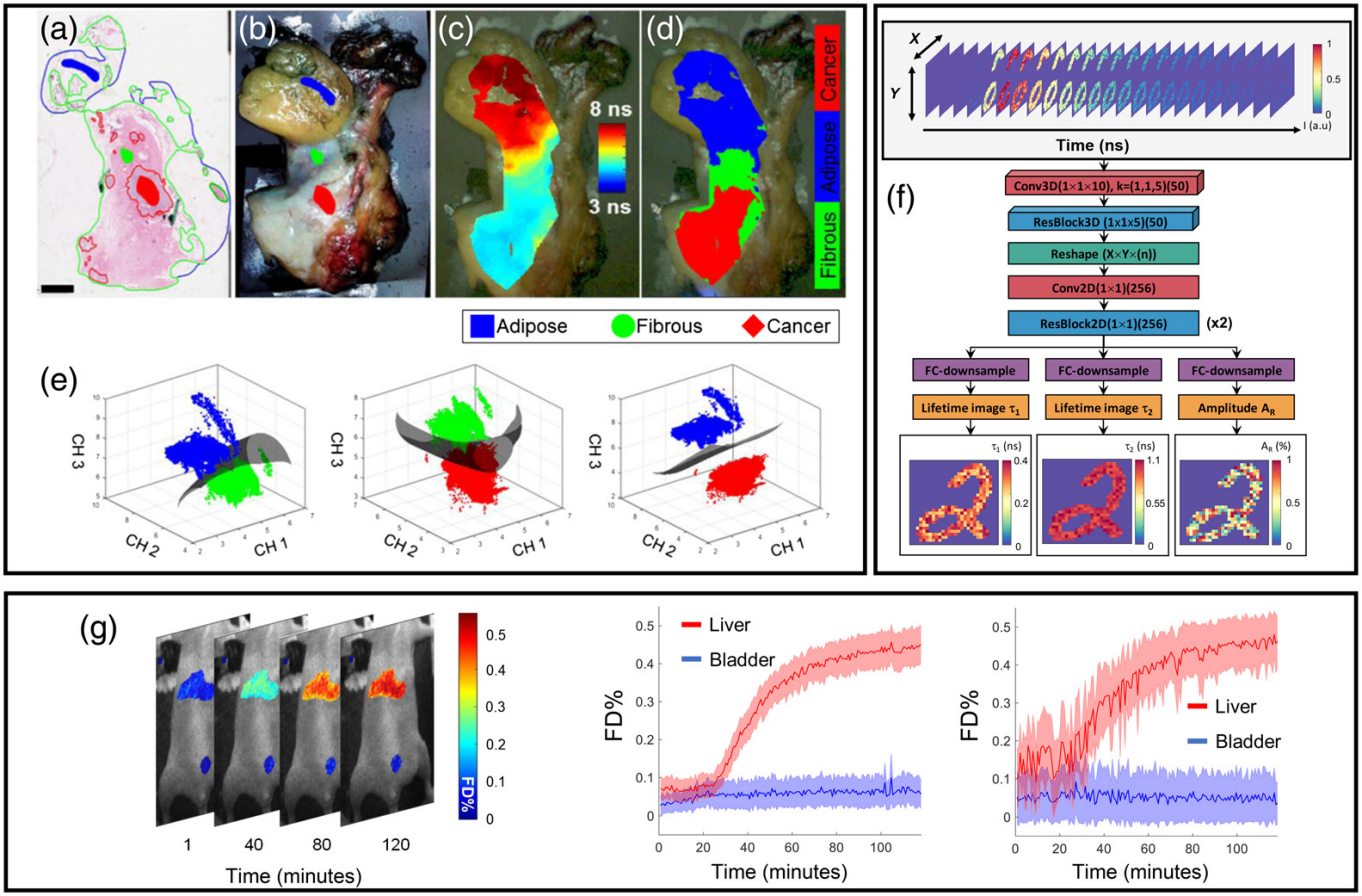
(a) Breast tissue samples labeled by histology and (b) corresponding white light image. The blue, red, and green markings are tracings added by the pathologist. (c) White light image augmented with fluorescence lifetime and (d) tissue classification results. (e) Example SVM hyperplane separations retrieved using FLI values retrieved across different spectral channels (reproduced with permission from Ref. 91). (f) DNN architecture of FLI-Net. (g) Example Förster Resonance Energy Transfer (FRET) fraction (FD%) results obtained via FLI-Net across two organs (liver and bladder) over the span of 2 h postinjection of transferrin. The FD% average and standard deviation across both ROIs over all acquisitions retrieved via FLI-Net (middle) and least-squares fitting (rightmost) (reproduced with permission from Ref. 92).

Given the high-sensitivity inherent to FLI, numerous studies exploring the technique’s capability with regards to classification *in vitro* have been undertaken within the last decade. Most recently, FLIM classifiers have been applied *in vitro* for label-free assessment of microglia^86^ and T-cell activation,^87^ as well as for exogenous labeling of intracellular components^88^ and monitoring of intracellular pharmacokinetics.^89^ In addition, ML classifiers have been used for FLIM-based tissue discrimination and characterization in applications including diagnosis of cervical precancer,^90^ breast cancer resection ^91^ [Figs. 3(a)–3(e)], and oropharyngeal margin assessment.^93^ However, this has been almost entirely relegated to microscopic or raster-scanning-based applications—technologies that are intrinsically limited in their (pre)clinical utility. In contrast, the potential applicability of wide field FLI extends to applications such as supremely sensitive fluorescence guided surgery and whole animal preclinical imaging, among others. It is precisely for applications of this type in which real-time analysis is paramount and the use of DL is positioned for great impact. Additional discussion on this topic can be found elsewhere.^94^^,^^95^

As in the previous section focusing on OPs retrieval, one significant development in leveraging DL models is not for classification tasks but to enable fast and fit-free estimation of lifetime parameters. Such prediction tasks are inherently far more challenging than classification tasks but are poised to greatly impact the FLI field by providing fast and robust tools that can be used by the end user communities while enabling reproducibility.

### 4.2 Deep Learning for Fluorescence Lifetime Image Reconstruction

Lifetime parameter estimation is typically performed by fitting a time series dataset (fast temporal decays) to (multi)exponential models. Wu et al.^86^ presented the first DL methodology for fluorescence lifetime image reconstruction, wherein the authors employed an MLP-based approach. The network deemed “ANN-FLIM” was trained with entirely simulated FLIM decays for biexponential FLIM reconstruction and benchmarked against an implementation of least squares fitting (LSF). The group reported an increased reconstructive performance via ANN compared with LSF. However, the authors reported a high rate of suboptimal convergence using LSF (4.07% of pixels), resulting in many dark spots that are completely unseen in the reconstruction obtained via MLP. This is a known problem of all iterative-fitting procedures, which rely heavily on the chosen input parameters and often converge at the upper or lower bounds. Further, the authors reported a 566-fold speed-increase over LSF (1.8s versus 1019.5s over a 400×400×57 FLIM voxel). Following on this work, Zickus et al.^96^ used an MLP trained with simulated data [Figs. 3(a) and 3(b)], in combination with image stitching, to retrieve a 3.6-megapixel (1875×1942  px) wide field FLIM image reconstruction using a time-resolved single-photon avalanche diode (SPAD) array. Similar to ANN-FLIM, Zickus et al. reported significant reconstruction speed improvements using an MLP (3.6 s) compared with conventional least-squares fitting (56 min). However, MLP is known to become unwieldy for high-dimensional data such as images, and they have been replaced by CNN in many computer vision applications.

Recently, Smith et al.^92^ presented a workflow for biexponential FLI (microscopy and macroscopy) reconstruction-based around a 3D-CNN trained with simulation data. Contrary to MLPs, where the objective is to map each temporal point spread function (TPSF) to a feature vector through a learned regression, the author’s 3D-CNN (deemed fluorescence lifetime imaging network, FLI-Net) was crafted to take large spatially resolved fluorescence decay voxels as input (x x y x t) and output concurrently three lifetime maps parameters, namely, $\tau_{1}\;(\mathrm{ns})$, $\tau_{2}\;(\mathrm{ns})$, and their relative abundance $A_{R}\;(\%)$, at the same spatial resolution as the input. Moreover, the network was made FCN, or capable of taking input of any spatial dimensionality (any image size). The authors validated FLI-Net’s capability to retrieve highly accurate FLI reconstruction across multiple FLI technologies (TCSPC and gated-ICCD) and applications (endogenous metabolic^97^ and Förster Resonance Energy Transfer [FRET]^98^ imaging). FLI-Net demonstrated high accuracy when tested with experimental data not used during the network training. Of importance, the network was validated for the NIR-range in which lifetimes are far shorter than the visible range and close to the temporal instrument response function, which is a very challenging case. Moreover, of significance, FLI-Net significantly outperformed the classical fitting approach in the case of very low photon counts. This is highly noteworthy for biological applications as fluorescence signals are dim, leading to relatively high-power illumination or long integration times for many applications. In turn, this can generate issues such as photobleaching or acquisition times incompatible with clinical applications.

Following on this seminal work, Xiao et al.^99^ introduced an alternative 1D-CNN architecture for FLIM reconstruction. In contrast to FLI-Net’s 3D architecture, which takes all FLI data voxels as input and outputs 2D images of the lifetime parameters, the 1D model was crafted to process each TPSF individually. The authors’ 1D-CNN, following a similar training strategy as laid out in Ref. 92, does not necessitate 3D convolution operations and thus offers the advantage of decreased computational burden. In particular, this simpler model is amenable to parallelization and to onboard integration. Such implementation should offer the opportunity for real time and robust FLI in low-cost settings. However, they are less suitable for use in applications that involve higher dimensionality. For instance, in the case of DOI applications, the OPs of the tissue may affect the fluorescence temporal data by attenuating (absorption/scattering) them and/or delay them (scattering) and hence should be considered. In his regard, Smith et al.^100^ expanded upon FLI-Net by augmenting time-resolved MFLI with SFDI-derived bulk OP information to enable OPs-corrected lifetime parameter estimation, as well as an estimation of the depth of fluorescence inclusions (referred to as topography of LiDAR). For this task, a Siamese DNN architecture was designed to take as input MFLI time decays and SFDI-estimated OP maps (i.e., absorption and scattering) and output both fluorescence inclusion depth and lifetime maps at the same resolution as the inputs. The data simulation workflow used for training was updated to mimic data acquired experimentally via Monte Carlo modeling of light propagation through turbid media.^101^ Overall, the proposed computational technique is the first of its kind to exhibit sensitive lifetime retrieval over wide bounds of scattering, absorption, and depth, with real-time applicability over large fields of view. It is expected to have great utility in applications such as optical-guided surgery.

Another area of growth in FLI imaging is the development of multispectral or hyperspectral lifetime imaging systems^102^ that promise to increase specificity by enable enabling to multiplex and/or unmix biomarkers signatures.^103^^–^^105^ However, accurate biological interpretation of the acquired rich data is often challenging due spectral crowding (highly overlapping emission spectra) and complex temporal features, especially in the case of $n_{\mathrm{coeff}}>2$ (tri-exponentials, FRET, etc.). Recently, Smith and Ochoa-Mendoza et al. proposed the use of DL along with a novel data simulation routine to optimize the inverse-solving procedure intrinsic to hyperspectral lifetime unmixing,^106^ which for the first time simultaneously used spectral and temporal contrast signatures. Building upon the simulation procedure used in FLI-Net, the group generated TPSFs across all wavelength channels—dictating intensity by spectral emission profiles chosen at random at each pixel. The DNN (named “UNMIX-ME”) mapped 4D TPSF voxels of size x×y×t×λ to n coefficient images, each one containing the unmixed abundance of the n fluorophore. Therefore, if n=3 then 3 abundance maps will be the network output, each one showing the abundance of components 1 to 3, respectively, with equivalent spatial dimensionality. UNMIX-ME outperformed the conventional sequential iterative fitting methodology by demonstrating higher *in silico* estimation accuracy of fluorophore abundance in the case of tri- and quadri-abundance species/states. Notably, the authors applied the model to small animal FRET quantification of transferrin^107^ and trastuzumab^108^ kinetics *in vivo* demonstrating its utility for DOI applications. Still, UNMIX-ME requires as inputs 4D data voxels (x×y×t×λ) that require high-end instruments, such as an hyperspectral single-pixel time-resolved camera.^109^ This system is dependent also on a inverse problem to generate the spatially and spectrally resolved maps, an inverse problem that can greatly benefit DL methodologies.

### 4.3 Deep Learning for Fluorescence Lifetime Image Formation—Single-Pixel Imaging

Pixelated cameras based on CCDs and CMOS technology have been widely employed for biomedical optics applications to directly acquire the pixelated image of the sample plane.^110^^,^^111^ Despite their multiple advantages, customizing them to detect at wavelengths outside silicon-based technology can prove complex. This can be further complicated by the need for hyperspectral and/or tomographic images or higher acquisition frame rates.^112^ In these cases, the usage of single-pixel imaging has been proposed as the arrangement can be accomplished with a single-detector offering superior performances.^112^ A single-pixel imaging setup is commonly composed of a spatial light modulator, such as a digital micromirror device (DMD), that can “structure” the sample’s emissions into a predetermined pattern before reaching a single detector (PMT, 1D-SPAD). Because the patterns are known, the image of the sample plane can be inverse solved from the collected emissions. The number of patterns traditionally equals the number of pixels in the image space; however, compressive sensing (CS) strategies have helped reduce the number of patterns needed for a determined resolution.^113^^,^^114^ In diffuse optics, the usage of patterns rather than raster scanning approaches allows for the use of higher illumination power as well as fields of view as large as the DMD space. Of note, the quality of the acquired data is highly dependent on the amount and type of patterns, the OPs of the sample plane, and the detector’s specifications.^115^

**Fig. 4:**
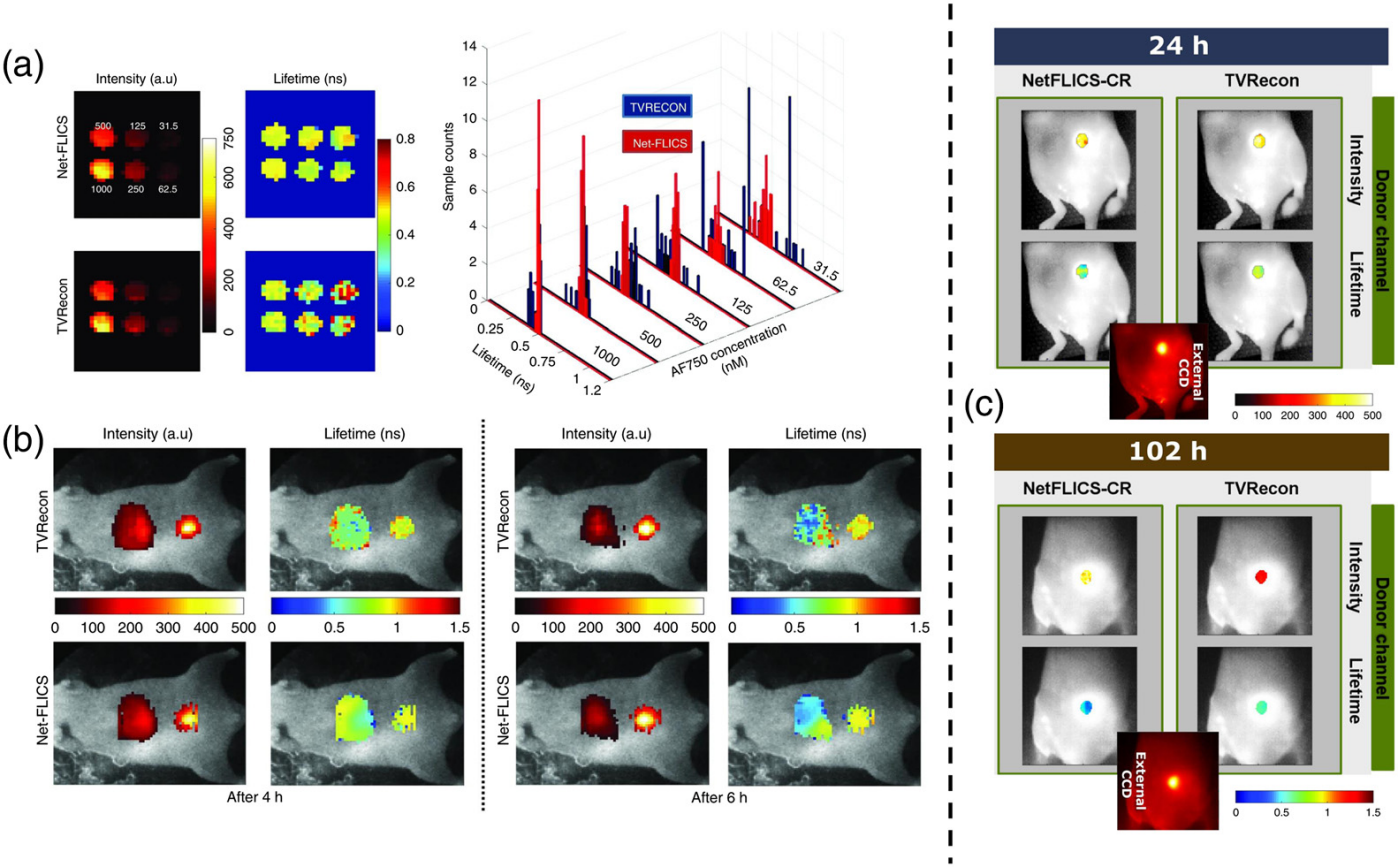
(a) NetFLICS reconstructed intensity and lifetime images from time resolved CS single-pixel raw data for an *in vitro* dye with decreasing concentration. (b) Quantification of Transferrin receptor-target engagement in liver and bladder areas.^17^ (c) Intensity and lifetime images of HER2 targeted tumor with the drug Trastuzumab as imaged 24 and 102 h postinjection.

For MFLI applications, single-pixel imaging has been implemented to obtain hyperspectral time domain (TD) data, which can be inverse solved into a 2D intensity image. In addition to the intensity profiles, each inverse solved pixel contains a respective TPSF that can be mono or multiexponentially fitted through a separate optimization algorithm to obtain a lifetime value per pixel.^9^ Therefore, single-pixel-based MFLI typically necessitates two steps: (1) use of an inverse solver to reconstruct the spatial image with temporal decays at each pixels and (2) subsequent use of a minimization algorithm for retrieval of lifetime per pixel to obtain the lifetime map(s). Yao et al.^17^ were the first to propose replacing this two-step process with a CNN capable of doing both tasks simultaneously. This CNN, named NetFLICS, takes as input the temporal curved acquired for each individual experimental pattern and outputs two images, one for fluorescence intensity and one for lifetime value. The authors reported that Net-FLICS outperformed the classical total variation reconstruction approach used in the field of single-pixel imaging in all conditions tested, including simulations, *in vitro* and *in vivo* [in Figs. 4(a) and 4(b)]. Moreover, NetFLICS was four orders of magnitude faster than the current inverse-based and fitting methodologies. Finally, and similar to FLI-Net, the FLI quantification provided by NetFLICS was superior under photon-starved conditions. However, NetFLICS was designed and trained to output 32×32  pixel images based on inputting data from 512 patterns (50% compression ratio of a 1024 Hadamard base). To increase image resolution and but also decrease experimental acquisition time, with a focus on *in vivo* settings, Ochoa et al.^113^ proposed NetFLICS-CR, which allows single-pixel TD data to be reconstructed to 128×128  pixel resolution intensity and lifetime images while only using 1% and 2% of the required data (99% and 98% data compression), which corresponds to 163 and 327 patterns out of 16,384 total Hadamard patterns. The significant data compression allowed for a reduction in experimental acquisition time from hours to minutes in *in vivo* settings. NetFLICS-CR architecture follows the two-branch design of NetFLICS as well as having the same functional blocks; however, it adds the usage of 2D separable convolutions and the compressed data training section. Figure 4(c) shows reconstructions for a Trastuzumab HER2 targeted tumor at two different timepoints as reconstructed for a 99% compression for a traditional inverse solved method based on TVAL3 solver and NetFLICS-CR, with the latter one being in accordance with the expected biological outcome. Of note, even though both NetFLICS and NetFLICS-CR were trained solely with single-pixel fluorescent MNIST-based^98^ simulated samples, they were capable of accurately reconstructing single-pixel experimental data in all conditions, even at extreme compression ratios.

As FLI found utility in an ever-increased numbers of applications and is becoming more widespread, DL is expected to greatly facilitate its acceptance by providing user-friendly tools that will permit standardization of the data processing pipeline and reproducibility of biological findings. It is expected that first the ubiquitous use of dedicated DL models will be adopted in analysis of FLI microscopy. This is supported by recent developments in open-sourced, user-friendly FLIM analysis software (e.g., FIJI’s FLIMJ^116^), which are amenable to implementation of DL-based features. Beyond speed, accuracy, and ease of use of FLI in low photon counts settings, DL is also expected to impact the instrumental and imaging protocol design associated with FLI. For example, Higham et al.^117^ trained a DCAN for real-time single-pixel imaging by incorporating binary pattern basis projection directly into their model’s encoder to learn optimal binary pattern sets for a wide range of RGB images. Further, recent work has demonstrated great strides in reducing MRI acquisition times using optimized sampling of k-space patterns.^118^^–^^120^ Hence, the incorporation and/or adaption of DL approaches to optimize both the acquisition protocol as well as the reconstruction performance of these technologies is expected. Further, DL will likely impact the workflow and eventual adoption of many notable MFLI applications, such as multiplexed preclinical imaging,^121^ and multimodal MFLI registration,^122^ among others.

## 5 Deep Learning for Diffuse Optics-Based Tomographic Imaging

Still, even if most of the abovementioned techniques/applications pertain to the field of DOI due to the diffuse nature of the light collected to image the sample, they are typically limited to shallow subsurface sensing and, hence, require relatively simple forward modeling methods. When deeper tissues are investigated and 3D capabilities are required, the inverse problem becomes more complex and challenging to solve. This field, referred to as DOT when focusing on the 3D mapping of OPs ($\mu_{a}$ and $\mu_{s}^{\prime }$), fluorescence molecular tomography (FMT) when attempting to retrieve the biodistribution of a fluorophore, and bioluminescence tomography when the molecular probe is bioluminescent, is hence dependent on the selection of dedicated forward models, refined inverse solvers, and regularization techniques. Due to the ill-posed and ill-conditioned nature of the inverse problem, the selection and optimization of these three important components of the inverse problem greatly impact the image reconstruction process in terms of computational burden, stability, and accuracy. This is still after three decades of being an expert domain. Hence, following the trend of investigating the potential of DL for image formation, there has been an increased interest in the application of DNNs for diffuse tomographic imaging with the goal of improving computational speed and user friendliness while enhancing the reconstruction quality.^123^^,^^124^ We provide the summary of current efforts for the three subfields of DOT in the next sections.

### 5.1 Deep Learning for Diffuse optical Tomography (Optical Properties Contrast)

**Fig. 5:**
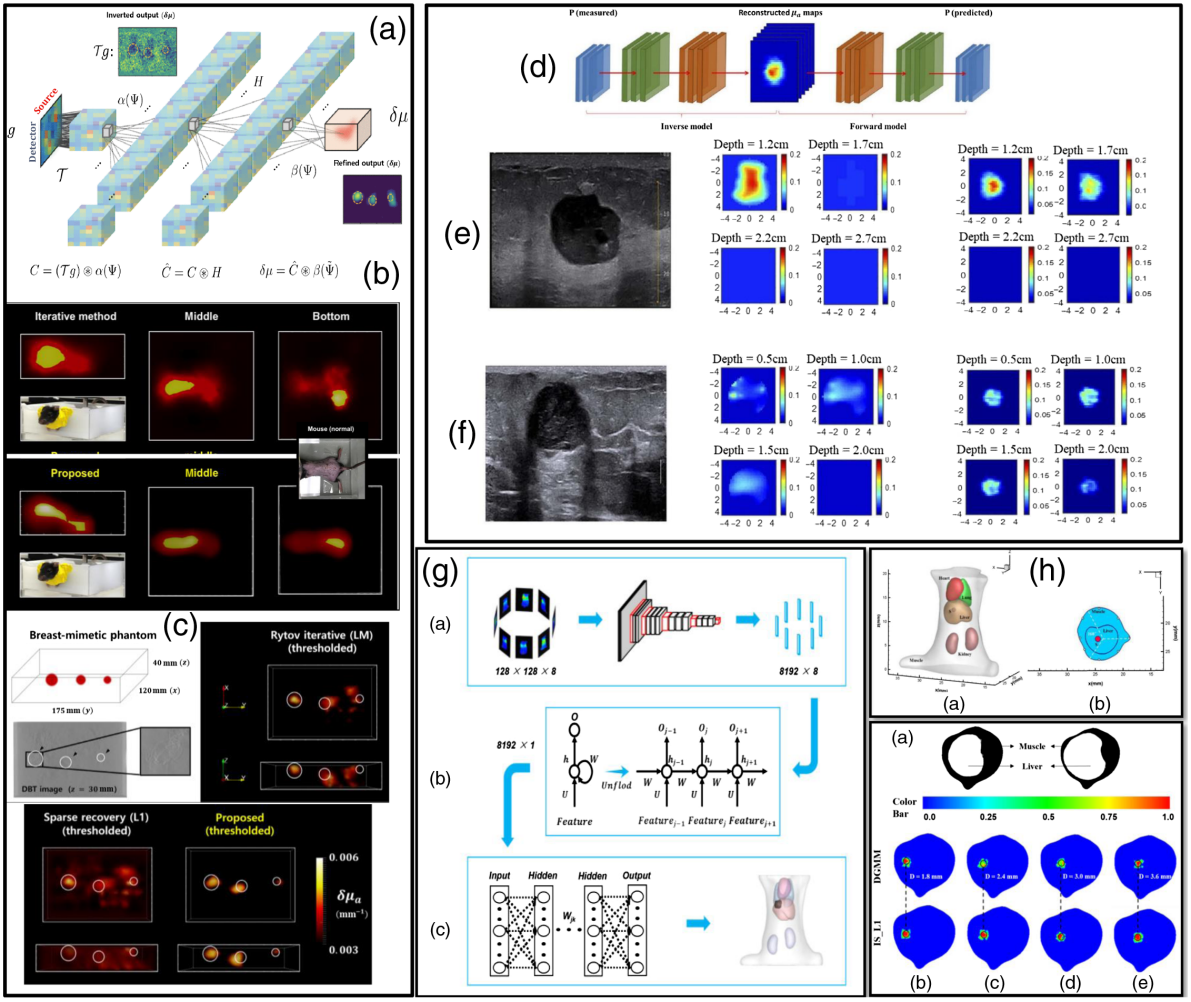
(a) Lippmann–Schwinger equation-based DNN.^123^ (b) *In vivo* mouse body as imaged with DNN versus the Rytov method. (c) Reconstructions for breast-mimetic phantoms versus inverse solved methods. (d) Full training architecture scheme as described in Ref. 125. (e) Results for example patient 1 (tumor 1.2-cm depth), and patient 2 (0.9-cm depth) in (f), are displayed with corresponding ultrasound image in first column, validation with the traditional Born-CGD reconstruction method shown in second column, and in the third column results for the proposed DL-based approach. (g) Reconstruction process for DGMM architecture. First-stage input and output matrices. GRU stage and last stage based on MLP. (h) 3D view of simulated mouse and organs and liver targeted with a source S as seen in the axial cross section. Axial cross-sectional DGMM reconstructions for sources at different depths from 1.8 to 3.6 mm.^126^

If DOT has been historically the first focus of 3D diffuse imaging, it is still not the main application for DL image reconstruction. Indeed, to date only a few works have been reported. Yoo et al.^123^ were the first to investigate the potential of a DL end-to-end model to reconstruct heterogenous optical maps in small animals. Their proposed DNN, shown in Fig. 5(a), followed a classical encoder–decoder structure that aims at solving the Lippmann–Schwinger integral equation with the deep convolutional framelets model^127^ by doing a nonlinear representation of scattered fields and avoiding linearization, or iterative Green’s functions calculations. The input data, i.e., light intensity surface measurements, are translated to the voxel domain, and unknown features are learned from the training data through a fully connected layer, which is followed by 3D convolutional layers and a filtering convolution. Their training data were simulated with NIRFAST^128^ with up to three spherical inclusions of different sizes (radii∈[2  mm,13  mm]) in which each voxel in their space had a defined set of OPs. DNN training used Adam optimizer with dropout and early stopping to avoid overfitting, and Gaussian noise filtering was applied to improve generality. The DNN was evaluated using biomimetic phantoms [Fig. 5(c)], in a healthy nude mouse [Fig. 5(b)] and in a mouse with a tumor. Despite the multiple accomplishments, the Lippmann–Schwinger approach requires a separate measurement from the homogenous background, which is unfeasible for clinical scenarios; therefore further research aims at solving this disadvantage. Despite this, the use of the Schwinger–Lippmann model to structure the neural network helped to remove the so named “black box” design uncertainty. Furthermore, due to this design, the nonlinear physics of the photon propagation and inverse solving of absorption contrasts can be reconstructed through DL, providing improved reconstruction for both *in vitro* and *in vivo* murine experiments. Nevertheless, the reconstruction speed in the millisecond range allows for fast *in vivo* imaging applications.

Following the autoencoder approach, the work of Ref. 125 aims to inverse solve the spatial distribution and absorption value of targets in both phantoms and clinically acquired data for breast cancer applications. Simulated and phantom acquired data were used for network training. The *in silico* data were simulated through the finite-element method. Data from measured homogeneous targets with no absorption contrast between them were used in the training set, whereas data from inhomogeneous targets with varying absorption within themselves were used in the validation set. COMSOL software was used as a forward modeling tool. Measurements were simulated from inclusions with varying radii, center depths, $\mu_{a}$ absorption, and reduced scattering coefficients $\mu_{s}^{\prime }$ to mimic layers similar to those of breast cancer tissue. *In vitro* and clinical data were acquired with an ultrasound guided DOT frequency-domain platform with 9-point sources and 14-point detectors.^129^ The autoencoder architecture uses two sections of neural networks. The first section reconstructs OPs from DOT measurements, but for this stage, the weights information for the trained second section (which involves a forward model that inputs the $\mu_{a}$ map ground truth and outputs the predicted perturbation) is used during training to improve accuracy. The second stage uses a loss function that reflects the MSE between input DOT measurement (which is the input of the first section) and the prediction. The first section inputs the DOT measurement and outputs the $\mu_{a}$ absorption map of the area, while using the MSE between $\mu_{a}$ ground truth and $\mu_{a}$ reconstruction plus weights from the second section. Finally, these sections were integrated into a single full architecture as shown in Fig. 5(d), using the individual section weights as initial estimates for a loss function that included a Born object function constraint and anatomical information (target radius and voxel distance) as obtained from the ultrasound image. The results from *in silico*, *in vitro*, and clinical information [examples provided in Figs. 5(e) and 5(f)] indicate that the proposed model accuracy is higher than traditional reconstruction methods such as Born-conjugate gradient descent, decreasing the mean percentage error from 16.41% to 13.4% in high-contrast targets and from 23.42% to 9.06% in low-contrast ones, while also showing improved depth localization. This was also true for the used clinical datasets, in which absorption contrasts were better estimated.

The work of Deng et al.^130^ extended on the AUTOMAP architecture^131^ shown in Fig. 1(a), where an FC layer inputs the data into an encoder–decoder structure that is followed by a U-Net arrangement for image denoising and quality improvement. Additionally, the model employs skip connections to retrieve and enhance high-resolution features for reconstruction. For training, photon propagation models are simulated through Monte Carlo extreme photon propagation modeling with single spherical inclusions that have randomly assigned OPs, positions, and sizes. The simulation included 48 sources and 32-point detectors as in an optical breast imager previously presented by the group. The first part of network (FC layer and encoder) was trained first, and the weights were fixed to then reset the learning rate to train the second section (U-Net layers). Furthermore, the proposed loss function for this work penalizes the inaccuracy from the inclusions rather than the whole volume, which greatly accelerated the training process. The approach was tested in comparison with an autoencoder-only network and conventional finite-element method. The proposed approach resulted in more accurate localization depth and tumor contrast compared with conventional approach for larger inclusions. This was also true for inclusions with smaller diameters, multiple inclusions, and irregular shapes, with exception of ≤5  mm inclusions with low contrast. Despite training on single inclusions, the CNN was able to generalize to multiple inclusion cases in millisecond reconstruction time, though further validation for experimental datasets must be tested. Additionally, the use of extrinsic probes that have higher fluorescence quantum yields and hence can greatly improve the signal-to-noise ratio has also been exploited. In this regard, fluorescent probes that target specific receptors in a tumor surface are employed to provide better contrast and localization. Despite the ongoing progress on probe design, reconstructing the spatial distribution and location of the targeted fluorescent areas suffers from many of the same challenges as we previously mentioned for reconstruction of optical contrasts. Herein DL approaches have also been applied to the reconstruction of fluorescence tomography.

### 5.2 Deep Learning for Fluorescence Molecular Tomography (Fluorescence Contrast)

Guo et al.^132^ proposed using an end-to-end DNN using a deep-encoder and decoder architecture (3D-En-Decoder) to perform the FMT reconstruction task. This 3D-En-Decoder learns the relationship of simulated fluorescent surface photon densities Φ originating from a deep-seated inclusion to directly output the estimated location and volume of the inclusion, without the need to model a Jacobian matrix. The network is composed of three main sections: a 3D-Encoder section that inputs Φ, a middle section containing an FC layer, and a 3D-Decoder yielding the output x. The 3D-Encoder section is composed of convolutional layers followed by batch normalization, ReLU activation, and max pooling, and it learns features of the acquired surface photon densities Φ regarding known parameters, including background OPs and the position and size of the fluorescence target(s). The 3D-Decoder section is composed of convolutional layers preceded by upsampling and followed by batch normalization and ReLU activation. This layer outputs the 3D distribution maps of the fluorophore spatial distribution. For training, FMT simulated sets were generated before the simulation/experimental tasks, with 10,000 samples for training and 1000 for validation. The simulations replicated a cylindrical sample imaged every 15 deg from 0 deg to 360 deg. The network was tested for simulated experiments with tube targets at different distances inside the medium, providing better 3D reconstruction accuracy and quality than the L1 regularized inverse solving method, which was also validated for an experimental phantom as quantified by CNR, Dice, LE1, and LE2 metrics. The 3D-En-Decoder reconstruction time was 0.23 s—in contrast to 340 s necessary via L1-regularized inverse solving.

Of note, the simulated training set matched the experimental settings accurately, as required in supervised learning. Another end-to-end DNN for FMT was proposed by Huang et al.^126^ DGMM operates with a “gated recurrent unit (GRU)” and MLP-based architecture. As shown in Fig. 5(g), segment one encodes feature information from the input, which is a 3D matrix composed of eight RGB images acquired at different views. Then a set of 13 convolutional layers that contain in between max pooling layers are followed by ReLU activation functions. The second stage uses GRU to combine the output features from the first stage into a single vector. The performance of GRUs is dependent on an “update gate,” which helps define the magnitude of information from prior time steps that should be passed through. In complement, a GRU’s “reset gate” decides how much information from prior states to forget. The output of the GRU is a fused 8192×1 feature vector as seen in Fig. 5(g). The output of the GRU is received by the last stage with an MLP to reconstruct the location of the fluorescent target as well as its overall shape. The MLP contains two hidden layers with dropout to aid overfitting followed by a ReLU activation. Simulated Monte Carlo samples of a mouse model and five of its organs are used for training, and a different *in silico* mouse model with a fluorescent target S in the liver [Fig. 5(h)] was reconstructed. Barycenter error indicated that single fluorophore S was correctly positioned with respect to ground truth with comparable results to L1 inverse solved reconstructions. This is also true for targets S with depths that varied from 1.8 to 3.6 mm [Fig. 5(h)]. This architecture provides the target’s location and not its 3D characteristics. Moreover, it has been validated with single inclusions and model-mismatch between the training model and test settings not investigated.

Another variation of FMT is mesoscopic tomography or MFMT,^133^ which provides higher depth and resolution than conventional FMT, reaching ∼100  μm resolution and up to 5-mm depth. For this application, DL has been proposed not to completely replace the traditionally inverse solving procedure but to enhance the depth localization and the clarity of the reconstructed images.^124^ The input of the network is the estimated fluorescence location and distribution as yielded by a traditional depth-dependent Tikhonov regularization; then a trained 3D CNN translates the regularization output into a binary segmentation problem that, when solved, will result in a reduction of the regularization reconstruction error. The network is composed of 5 convolutional layers with zero padding and 2 FC layers with ReLu activation, and the training datasets consisted of 600 randomly generated ellipsoids and balls to reconstruct simplistic GRUb geometric figures (e.g., rectangular prisms, spheres, and ellipsoids). This work highlighted the potential to reduce the volumetric reconstruction and fluorophore localization error while increasing intersection over union of the reconstructions by 15% with respect to ground truth. However, Tikhonov regularization is time-consuming; therefore, an optimized first step would improve the current workflow. Yang et al. built upon this previous research in Ref. 134. Here DL is used as a complementary method to accelerate reconstruction time and quality, while employing a conventional inverse solving algorithm, in this case, the least-squares inverse solver with weighted $L_{1}$ norm. In this work, the Jacobian sensitivity matrix (forward modeling) is accomplished by Monte Carlo-based simulations. A symmetric CNN is then used to find the principal components of this sensitivity matrix so that the size of the final Jacobian used for inverse solved reconstruction can be reduced. This allows for artifact suppression as the noise of secondary components in the Jacobian will be removed; furthermore if the Jacobian size is reduced, the computational time and burden to inverse solve the 3D target distributions will also be reduced. The symmetric network follows an encoder–decoder architecture through convolutional and deconvolutional blocks with ReLU activation functions. Training is performed with MSE loss and SGD as an optimizer. The results of this work demonstrate that, when using the proposed network, the Jacobian size can be reduced from 21168×6615 to a matrix of size of 6400×6615, yielding more accurate and faster resolved 3D fluorescence reconstructions. The approach was tested *in silico* and for synthetic vasculature samples, yielding better results than when using an inverse solving process alone without any reduction, both in reconstruction accuracy and speed.

Despite DL being successfully used as a complementary tool to the traditional reconstruction process, many of the highlighted works aim to accomplish an end-to-end reconstruction solution through DL. For example, the work of Nizam et al.^135^ also addressed on the use of an AUTOMAP-based architecture for end-to-end recovery of fluorescence targets for k-space-based fluorescence DOT. This work assumes a reflectance imaging configuration with wide field structured illumination and wide field detection. This is important as it can provide faster image recovery than conventional raster scanning approaches. For this work, the CNN follows a similar architecture as the AUTOMAP network with three fully convolutional/connected layers, especially because AUTOMAP was made for k-space modulated information acquired by MRI. For training data generation, first, the photon propagation model for a homogenous area with fixed dimensionality was simulated using MCX software,^136^ accounting for the k-space illumination patterns. Then EMNIST^137^ characters are voxelated and multiplied to the simulated homogenous Jacobian model to generate a measurement vector approximation. The usage of EMNIST-based simulations should provide better approximation to nongeometric tumors. This is a difference from the previously mentioned approaches that employ geometric structures such as spheres or circles for dataset simulation. The network inputs the one-dimensional measurement vector and aims to output the 3D distribution of the fluorophore. The approach was validated *in silico* and compared with traditional inverse solver methods such as LSQR and TVAL3-based reconstruction. The test samples included variations of letter embeddings from 2 to ∼8  mm depth, with comparable results to TVAL and LSQR-based inversion on shallow depths. However, once at higher depths, the CNN performed better than the compared approaches with more accurate localization and dimensionality reconstructions. This was also true for cases in which there are multiple letter embeddings. Further extension of the approach involves using a large range of varying OPs within the embeddings and validation for experimental datasets.

### 5.3 Deep Learning for Bioluminescence Molecular Tomography (Bioluminescence Contrast)

Bioluminescence is another class of the diffuse tomographic inverse problem. It pertains to the “inverse source problem”; inverse problems are notoriously extremely challenging in scattering media. Gao et al.^138^ recently reported on MLP model to obtain the bioluminescence density captured at the surface of the sample. The density of surface photons is the input for the first layer, which is adapted to the number of nodes at the surface of a standardized 3D mesh of a segmented mouse head. The mesh results from CT and MRI images and is used to describe the light propagation model at the brain region. Subsequently the network contains four hidden layers, each one proceeded by a ReLU activation and dropout. The units in these layers equal the number of nodes in the brain region, which are also used for the output layer, which yields the photon density of the bioluminescent source. The results displayed better tumor localization in comparison with the traditional fast iterative shrinkage/threshold (FIST) approach. The results from the used mouse models and the *ex vivo* analysis reinforced the accuracy of the proposed network in localizing tumors. However, further work involves the addition of a section that can reconstruct the tumor morphology and the tumor position, as well as the inclusion of a larger training set that covers a wide range of tumor variations. Further investigation of the generality of the network for tumor types that differentiate from the training sets is also necessary. The use of DL as an inverse solver is expected to allow for more accurate representations of the photon propagation model and the specific photon densities after tissue and fluorophore scattering. Depending on the quality of training data, the assumption of linearity and the use of extra constraints can be alleviated in comparison with the currently used method. After the inverse solving process, DL could be useful for segmenting regions of interest from the retrieved tomographic rendering. Moreover, these characteristics might be applicable to a variety of optical tomography applications.

## 6 Conclusions

The versatility of DL in DOI is unparalleled as it has been demonstrated to enable sensitive discrimination between tissue subtypes, fluorescence image reconstruction, optical parameter estimation—the list seemingly increasing *ad infinitum*. DL’s inherent benefits also permit investigators to significantly increase the feedback throughput and will aid in the translation of many techniques to the clinic. The generality illustrated by many of these tools provides promise that each usage can be context-dependent for each investigation and can yield results that match current gold standards in various fields, leading scientific investigation in new horizons previously thought unattainable. Looking forward, DL has great potential to be used for bridging the gap between experimental data acquisition and data analysis by providing the computational speed necessary for real-time applications. When optimized, these techniques will have a lasting impact on many regions beyond the developed world currently without access to high-power computational resources. Additionally, modifications to these neural networks can be made to inject principles of mathematics and physics directly through the means of custom loss functions. Custom loss functions permit the networks to use the principles of the subfields that they are in and to guide their mappings to solution spaces that can be expected from their corresponding problems.

Developments within DL explainability and interpretability, aiming to address the “black box” nature of high-performing models, will be critical for future widespread acceptance of these methods.^139^^–^^142^ Although there exists some skepticism about the validity of DL at present, dedicated efforts continue to provide greater degrees of insight into countless DL workflows with every passing day. With the current deluge of new techniques such as GANs, RL, and countless others, further development of new architectures as well as methods to ensure generalizability of such models will only continue to improve. Altogether, these developments will pave the way toward a future with DL as a reliable tool to accelerate and advance both the expansion of biomedical knowledge and the degree of care in clinical applications.
